# Alteration of brain functional networks induced by electroacupuncture stimulation in rats with ischemia–reperfusion: An independent component analysis

**DOI:** 10.3389/fnins.2022.958804

**Published:** 2022-08-03

**Authors:** Si-Si Li, Xiang-Xin Xing, Xu-Yun Hua, Yu-Wen Zhang, Jia-Jia Wu, Chun-Lei Shan, Mou-Xiong Zheng, He Wang, Jian-Guang Xu

**Affiliations:** ^1^School of Rehabilitation Science, Shanghai University of Traditional Chinese Medicine, Shanghai, China; ^2^Center of Rehabilitation Medicine, Yueyang Hospital of Integrated Traditional Chinese and Western Medicine, Shanghai University of Traditional Chinese Medicine, Shanghai, China; ^3^Department of Traumatology and Orthopedics, Yueyang Hospital of Integrated Traditional Chinese and Western Medicine, Shanghai University of Traditional Chinese Medicine, Shanghai, China; ^4^Institute of Science and Technology for Brain-Inspired Intelligence, Fudan University, Shanghai, China; ^5^Engineering Research Center of Traditional Chinese Medicine Intelligent Rehabilitation, Ministry of Education, Shanghai, China

**Keywords:** ischemic stroke, electroacupuncture, motor function, independent component analysis, resting state networks

## Abstract

Motor dysfunction is the major sequela of ischemic stroke. Motor recovery after stroke has been shown to be associated with remodeling of large-scale brain networks, both functionally and structurally. Electroacupuncture (EA) is a traditional Chinese medicine application that has frequently been recommended as an alternative therapy for ischemic stroke and is reportedly effective for alleviating motor symptoms in patients. In the present study, the effect of EA on the alterations of functional resting state networks (RSNs) was explored after middle cerebral artery occlusion/reperfusion (MCAO/R) injury using resting-state functional MRI. Rats were randomly assigned to three groups, including the sham group, MCAO/R group and MCAO/R+EA group. The ladder rung walking test was conducted prior to and after modeling to assess behavioral changes. RSNs were identified based on the independent component analysis (ICA) performed on the fMRI data from groups. EA treatment effectively reduced the occurrence of contralateral forelimb foot faults. Furthermore, our results suggested the disrupted function of the whole-brain network following ischemic stroke and the modulatory effect of acupuncture. The sensorimotor network (SMN), interoceptive network (IN), default mode network (DMN) and salience network (SN) were related to the therapeutic effect of EA on stroke recovery. Collectively, our findings confirmed the effect of EA on motor function recovery after cerebral ischemia reperfusion and shed light on the assessment of EA intervention-induced effects on brain networks. This study provides neuroimaging evidence to explain the therapeutic effects of EA in ischemic stroke and will lay the groundwork for further studies.

## Introduction

Stroke can be categorized into ischemic and hemorrhagic stroke, ischemic stroke accounts for approximately 80% of cases, and hemorrhagic stroke accounts for 20% (Wang et al., 2017). Ischemia stroke refers to localized ischemic necrosis or softening of brain tissues caused by permanent or temporary artery occlusion in the cerebral bloodstream, and it has the characteristics of high morbidity, high mortality, and high disability (Zhang et al., 2020; Guo et al., 2021). As a main cause of disability in adults, ischemic stroke can lead to long-lasting neurological deficits, motor and cognitive dysfunctions that have a considerable negative impact on patients’ quality of life (Lu et al., 2017). Therefore, elucidation of the pathological mechanisms underlying ischemic stroke has important implications for functional recovery after stroke.

Brain plasticity or neural plasticity can be defined as the capacity of the brain to modify the organization of the brain structure and function in response to new stimuli or environmental exposures (Jiang et al., 2015). Resting-state fMRI (rs-fMRI) is a powerful neuroimaging technique that has become a non-invasive method for studying brain function in patients suffering from ischemic stroke who suffer from motor disturbance or cognitive impairment (Saad et al., 2013; Chen et al., 2016). Previous studies have focused on the metrics that reflect regional spontaneous neuronal activity, such as regional homogeneity (ReHo) and the amplitude of low-frequency fluctuation (ALFF), which have been widely used to investigate brain functions following ischemic stroke (Liang et al., 2020; Hu et al., 2021). The advent of rs-fMRI has enabled studies to unravel brain networks involving many regions across different modalities, including white matter tracts, gray matter volume, and functional connectivity (Van Kesteren and Kievit, 2021). Large-scale brain networks have been used extensively in the study of neuropsychiatric and neurodegenerative disorders, such as depression, mild cognitive impairment, schizophrenia, Alzheimer’s disease and stroke (Williamson and Allman, 2012; Li et al., 2020). A wide network of the brain has been implicated in the recovery of motor function after acute ischemic stroke (Cheng et al., 2021). The resting state networks (RSN) refer to functional networks of brain regions that are active without a specific task or stimulus (Jeong et al., 2012), which comprise the sensorimotor network (SMN), interoceptive network (IN), default mode network (DMN), dorsal attention network (DAN), executive control network (ECN), salience network (SN), and primary sensorimotor, visual, and auditory network (PN) (Raichle, 2011). Independent component analysis (ICA), a data-driven approach, has been extensively used to identify multiple RSNs and investigate functional activities within and between brain networks *in vivo* (Huang et al., 2020). Focal cerebral ischemia leads to abnormal functional brain networks in resting-state conditions. How the different brain areas interact in stroke patients remains unknown. Research on the pathogenesis of ischemic stroke from the perspective of RSNs can reveal the underlying mechanisms of brain network reconfigurations, and it also has constructive significance for finding a suitable rehabilitation method.

Electroacupuncture (EA) is a type of therapy that applies a pulsating electrical current to acupuncture needles, thus enhancing the effects of acupuncture stimulation on acupoints (Xiang et al., 2019). It has been used in the treatment of various neurological diseases, such as stroke, Parkinson’s disease, epilepsy, and spinal cord injury (Zhao et al., 2019). A previous fMRI study found that acupuncture could play a role in ameliorating brain dysfunction and could lead to specific alterations in several resting state networks of sleep deprivation subjects (Dai et al., 2012). Another study found that the architecture of the whole-brain functional network was altered after acupuncture in healthy subjects, and the alterations certified the specificity of acupoints (Han et al., 2020). The clinical applicability and efficacy of acupuncture in rehabilitation after stroke has been demonstrated in several studies (Lv et al., 2021; Zhang et al., 2022). The Zusanli (ST36) and Quchi (LI11) acupoints are the most commonly used acupoints for the treatment of stroke (Xie et al., 2013; Liu et al., 2016a).

The purpose of this study was to investigate the disrupted function of the whole-brain network following middle cerebral artery occlusion and reperfusion (MCAO/R) and the therapeutic effects of EA at the LI11 and ST36 acupoints in a rat model. Here, we hypothesize that EA at LI11 and ST36 may ameliorate MCAO/R injury-induced motor function impairment by activating motor-related brain networks.

## Materials and methods

### Animals

**FIGURE 1:**
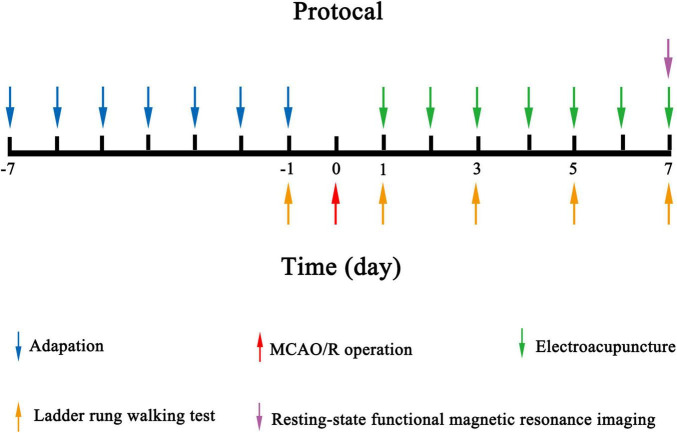
Experimental design used in the study.

Sprague–Dawley rats (clean grade, with a body weight of approximately 250–280 g) were provided by the Shanghai Laboratory Animal Research Center (Shanghai, China). Estrogen is known to be protective against ischemia–reperfusion injury; thus, female rats were not used in the current study to avoid the confounding effect of estrogen (Wang et al., 2019). Rats were housed under controlled lighting (12-h light/12-h dark cycle), humidity (40–50%) and temperature (23 ± 2°C) conditions. Rats were given *ad libitum* access to food and water. All animal experiments were approved by the Committee on Animal Care and Usage of Shanghai University of Traditional Chinese Medicine (Approval No. PZSHUTCM200110002). The National Institutes of Health Guide for the Care and Use of Laboratory Animals was strictly adhered to throughout this study. Twenty-four rats were randomly assigned to three groups: the sham group, MCAO/R group and MCAO/R+EA group. The experimental flow is presented in Figure 1.

### Focal cerebral ischemia reperfusion model

The rats were anesthetized with 3% pentobarbital sodium (30 mg/kg, intraperitoneal). The MCAO model was implemented according to the method reported by Longa et al. (1989). Briefly, the left common carotid artery, internal carotid artery (ICA) and external carotid artery (ECA) were exposed and isolated carefully. After distal ligation of the ECA, a small incision was made on the ECA, and a monofilament nylon suture (L3600, Jialing Co. Ltd., Guangzhou, China) was inserted from the stump on the ECA into the internal carotid artery until a slight resistance was felt. After a 2-h ischemia period, reperfusion was achieved by slowly removing the monofilament nylon suture to restore blood supply to the MCA territory. In the sham group, the rats underwent the same operation without insertion of the monofilament nylon suture. Successful model establishment was confirmed by the observation of rats failing to extend right forepaw, circling to the right, or even falling to the right (Longa et al., 1989).

### Electroacupuncture intervention

Electroacupuncture was performed at the same time of day (approximately 9:00 a.m.). The rats were fixed in an immobilization apparatus, with their bodies immobilized, leaving the head and limbs to move freely. Rats were acclimated to the immobilization apparatus at least 3 days prior to acupuncture intervention to relieve anxiety. In this experiment, 0.25 × 13 mm disposable sterile stainless-steel needles were inserted into LI11 and ST36. LI11 is located in the depression lateral to the anterior aspect of the radius joint of the forelimb, and ST36 is located 5 mm beneath the capitulum fibulae and lateral posterior to the knee joint on the contralateral side (Li et al., 2022). EA treatment was initiated on the first postoperative day and conducted for 30 min once a day for 7 consecutive days, with a frequency of sparse and dense waves of 2/15 Hz (HANS-200, Nanjing Jisheng Medical Co., Ltd., Nanjing, China). The intensity of the current was determined by observing slight jitter of the muscle.

### Ladder rung walking test

The ladder rung walking test is a locomotor test used to assess balance and coordination control of the forelimb and hindlimb. In the test, the rats were placed on one side of the ladder and required to walk on a horizontal ladder with irregular spacing (1–3 cm apart) three times. The total number of steps and the number of errors of the paralyzed forelimb were recorded. Foot fault (%) = (the number of wrong steps/total steps) × 100% (Metz and Whishaw, 2002).

### fMRI image acquisition

Functional MR images of brain were acquired from all rats in the resting-state using an 11.7 T animal scanner (Bruker Corporation, Germany) equipped with a surface coil (Bruker) at 1 week after surgery. After anesthetization with 5% isoflurane, the rats were fixed in the scanner. Anesthesia was continuously delivered (1.5% isoflurane combined with 0.05 mg/kg dexmedetomidine) throughout the entire scanning session, and a breathing machine was used to monitor the respiration of the rats. Rs-fMRI was acquired using an echo-planar imaging (EPI) sequence with the following parameters: flip angle = 90°, slice thickness = 0.3 mm, number of averages = 1, repetition time (TR) = 3,000 ms, echo time (TE) = 8.142 ms, and field of vision (FOV) = 27 × 27 mm^2^. After data acquisition, the image quality was visually checked immediately. Low-quality images were discarded, and an additional scan was applied.

### fMRI data preprocessing

The MATLAB statistical parametric mapping 12 (SPM12) toolbox^1^ was used for data preprocessing. All images in DICOM format were converted to NIfTI format, and the first 10 time points of the functional images were deleted to minimize the effect of instability MRI signals. Images underwent slice timing correction, coregistration, and realignment for head motion correction. Non-brain tissue was subsequently removed using MRIcron.^2^ All scans were manually realigned according to the anterior-posterior commissure. Subsequently, images were spatially normalized into the standard template and resampled to 2.06 × 2.06 × 2 mm^3^. Finally, images were smoothed with a full width at half maximum triploid as the voxel size (6.18 × 6.18 × 6 mm^3^) to increase the signal-to-noise ratio. Temporal bandpass filtering (0.01–0.1 Hz) was further performed to decrease the effects of low-frequency drift and high-frequency physiological noise.

### The intranetwork alteration of resting state networks

The preprocessed data of the three groups were merged and analyzed as one group. Spatial ICA was conducted for all preprocessed data using GIFT software.^3^ The proposed method consists of three major steps: dimension reduction via principal component analysis (PCA), ICA decomposition, and back-reconstruction for individual-level components. In this study, the data were first subjected to dimensionality reduction using the two-level PCA method. Then, independent component estimation was conducted using the information maximization (Infomax) algorithm. The independent components (IC) number was determined to be 20 based on previous studies and the minimum description length criteria (Calhoun et al., 2001; Hutchison et al., 2010). The InfoMax algorithm was used to decompose the data into 20 components. To achieve robust outcomes, this analysis was replicated 100 times. Group-level ICs were then back-reconstructed for each subject, and each estimated component received subject-specific spatial maps and time courses. To obtain voxel values comparable across participants, the ICA-determined networks were converted into Z-maps using Fisher Z transformation before entering group statistics (Song et al., 2011). Spatial maps for each of the RSNs were transformed to z values. A voxel-wise two-sample *t*-test was used to compare the differences between two groups.

### Statistical analysis

Statistical analysis was performed with SPSS 22.0 software (SPSS Inc., Chicago, IL, United States), and the results are expressed as the means ± standard errors of the means (SEM). Multiple group comparisons were analyzed by one-way analysis of variance (ANOVA) followed by the least significant difference test (LSD) when variances were homogeneous. Values of *P* < 0.05 were considered statistically significant. Pearson’s linear correlation coefficient was calculated to explore the relations between behavior and brain metrics, which were caused by cerebral ischemia/reperfusion injury and EA therapy. The significance threshold was set at *P* < 0.05, two-tailed.

## Results

### Electroacupuncture treatment significantly reduced the occurrence of forelimb foot fault

**FIGURE 2:**
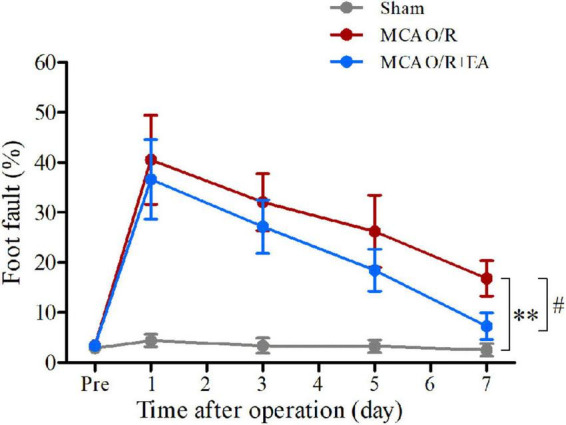
Treatment with EA reduces the occurrence of contralateral forelimb foot faults after MCAO/R. At 7 days after modeling, MCAO/R significantly increased the incidence of contralateral forelimb foot faults. The incidence of contralateral forelimb foot faults in the MCAO/R+EA group was decreased compared with that in the MCAO/R group at 7 days postsurgery. The data are presented as the means ± SEMs (*n* = 8 per group). ^**^P < 0.01 compared with the sham group, ^#^*P* < 0.05 compared with the MCAO/R group.

The ladder rung walking test results are displayed in Figure 2. The incidence rate of foot faults in the MCAO/R rats remained higher on the 7th day after the operation compared with the sham group (*P* < 0.01). The incidence of forelimb foot faults in the EA group was significantly decreased compared with that in the MCAO/R group on the 7th day after the operation (*P* < 0.05).

### Intra-network alterations in identified resting state networks in middle cerebral artery occlusion/reperfusion rats and the effect of acupuncture on resting state networks

**FIGURE 3:**
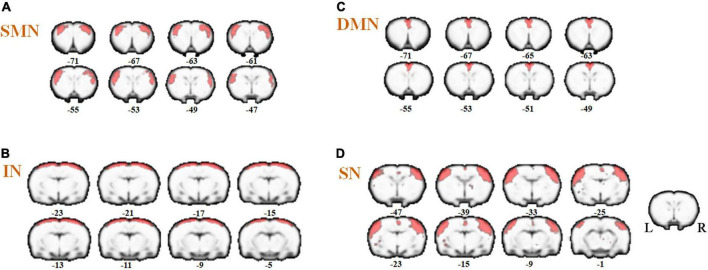
Coronal views of spatial maps for each resting-state network. **(A)** sensorimotor network (SMN), **(B)** interoceptive network (IN), **(C)** default mode network (DMN), and **(D)** salience network (SN). Each RSN map was obtained using a one-sample *t*-test across all individual IC patterns (FWE, *P* < 0.05). The right hemisphere of the brain is on the right side of the image.

**FIGURE 4:**
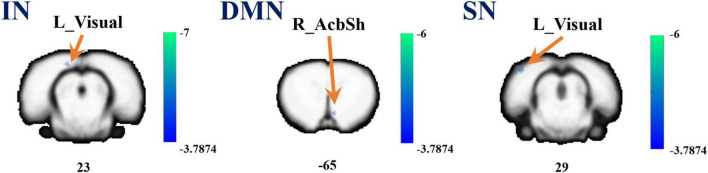
A brain with altered function within the network between the sham and MCAO/R groups. Altered functional activity in the interoceptive network (IN), default mode network (DMN), and salience network (SN). Warm colors denote higher functional activity in the MCAO/R group compared with the sham group, and cool colors denote lower functional activity in the MCAO/R group. Two sample *t*-test (*P* = 0.001, alphasim corrected, cluster size > 10).

**FIGURE 5:**
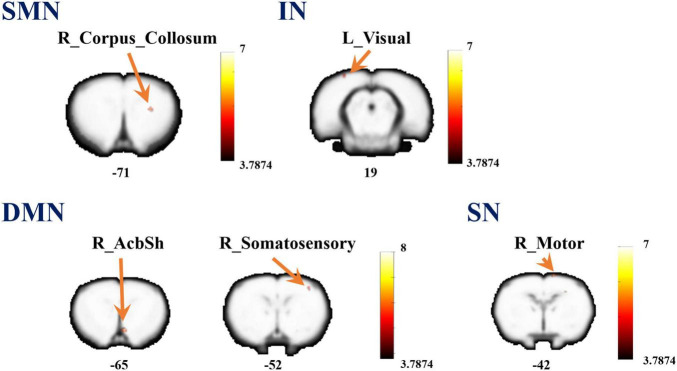
A brain with altered function within the network between the MCAO/R group and MCAO/R+EA groups. Altered functional activity in the sensorimotor network (SMN), interoceptive network (IN), default mode network (DMN), and salience network (SN). Warm colors denote higher functional activity in the MCAO/R+EA group compared with the MCAO/R group, and cool colors denotes lower functional activity in the MCAO/R+EA group. Two sample *t*-test (*P* = 0.001, alphasim corrected, cluster size > 10).

Referenced to the research of Dusica Bajic and colleagues (Bajic et al., 2017), four RSNs were identified, including the SMN, IN, DMN, and SN (Figure 3). We found significant changes of FC in these networks, three of these networks exhibited a decrease in the MCAO/R group compared with the sham group. In the IN, the activity of the left visual cortex was significantly decreased. In the DMN, the activity of the right nucleus accumbens shell (AcbSh) was decreased. In the SN, the activity of the left visual cortex was significantly decreased (Figure 4 and Table 1). We then investigated the effect of EA on these networks separately. These networks exhibited an increase after EA treatment (Figure 5 and Table 2). In the SMN, the activity of the right corpus callosum was increased. In the IN, the activity of the left visual cortex was significantly increased. In the DMN, the activities of the right AcbSh and somatosensory cortex were increased. In the SN, the activity of the right motor cortex was significantly increased.

**TABLE 1:** Differences between MCAO/R and sham groups in resting state functional connectivity of networks.

				MNI coordinates		
RSNs	Region label	Extent	*t*-value	x	y	z
IN	L_Cortex_Visual	10	–6.598	–17	26	23
DMN	R_AcbSh	10	–5.261	9	–26	–65
SN	L_Cortex_Visual	17	–5.456	–50	22	29

**TABLE 2:** Differences between MCAO/R and MCAO/R+EA groups in resting state functional connectivity of networks.

				MNI coordinates		
RSNs	Region label	Extent	*t*-value	x	y	z
SMN	R_Corpus_Collosum	19	5.275	32	3	–71
IN	L_Cortex_Visual	11	4.774	–34	36	19
DMN	R_AcbSh	19	7.427	7	–26	–65
	R_Cortex_Somatosensory	10	5.659	44	28	–53
SN	R_Cortex_Motor	14	6.353	16	44	–43

### Correlation analyses demonstrated a correlation between behavior and brain area functional activations

**FIGURE 6:**
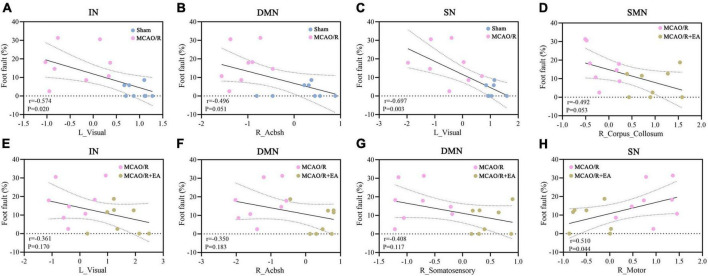
Correlation analyses demonstrate the link between behavior and brain area functional activations. **(A–C)** In the sham group and MCAO/R group, the behavioral results showed a significant negative correlation with the left visual cortex in the IN **(A)** and SN **(C)** networks. The rate of foot faults did not correlate (positive or negative) with the right AcbSh in the DMN **(B)**. **(D–H)** In the MCAO/R group and MCAO/R+EA group, there was a significant positive correlation between the rate of foot faults and right motor cortex area in the SN network **(H)**. The foot fault rate was not correlated (positive or negative) with the right corpus callosum in the DMN **(D)**, the left visual cortex in the IN **(E)**, or the right AcbSh in the DMN.

The BOLD signal values of brain regions that showed significant differences were calculated to analyze the Pearson linear correlations with the behavioral indices of foot fault. As shown in Figure 6, in the MCAO/R vs. sham groups, we found a significant negative correlation between the rate of foot faults and the BOLD signal value of the left visual cortex area in the IN network (*r* = –0.574, *P* = 0.02). In addition, the foot fault rate showed a significant negative correlation with the left visual cortex area in the SN network (*r* = –0.697, *P* = 0.003). The foot fault rate did not correlate (positive or negative) with the BOLD signal values of brain regions in the DMN. In the MCAO/R+EA vs. MCAO/R group, a significant positive correlation was found between the foot fault rate and the BOLD signal value of the right motor cortex area in the SN network (*r* = 0.51, *P* = 0.044). No regions in the other networks had functional activity that was positively or negatively correlated with the rate of foot fault.

## Discussion

Ischemic stroke is a serious central nervous system disease that may subsequently lead to loss of locomotor and cognitive function. EA is a therapeutic strategy to repair brain injury and improve functional outcome in acute ischemic stroke (Liu et al., 2009). As EA shows a beneficial effect in ischemic stroke, further investigation is needed to better understand the mechanisms of EA in ischemic stroke. In the current study, the ladder rung walking results demonstrated improved motor function of the paralyzed forelimb after EA treatment. Furthermore, this study explored brain networks involved in the potential modulatory effect of EA in rats with cerebral ischemia–reperfusion injury. LI11 and ST36 were chosen to explore the mechanisms underlying alterations in brain network connections after EA treatment. The SMN, IN, DMN, and SN were identified to be related to the therapeutic effect of EA on stroke recovery. To our knowledge, we are the first to explore the mechanisms underlying alterations in brain network connections after EA at the LI11 and ST36 acupoints in an MCAO/R model, as well as the correlation between behavior and brain activity.

fMRI has been a feasible tool to investigate brain changes following acupuncture in rats *in vivo*. The majority of published studies have focused on plastic changes in specific regions of the brain. Recently, studies have explored the relationships among brain structure, brain function, and behavior from the perspective of network scales. Connectivity between the ventral SMN and the ipsilesional frontoparietal network (FPN) was decreased in chronic severe stroke patients, suggesting that the control of motor function may be disrupted by the impaired cognitive control of the ipsilesional FPN to SMN (Zhao et al., 2018). The complex anatomical structure of each RSN is directly related to special brain functions. The SMN comprises the primary somatosensory cortex, motor cortices, and insular cortex (Kajimura et al., 2020). The SMN has been proposed to serve a variety of functions, such as sensorimotor integration, executive control, and emotional regulation (Yeo et al., 2011). The IN is a network containing areas of the anterior cingulate cortex and insula. The insula functions as the key switching center for processing and modulating pain, visceral sensory, emotion, and maintaining homeostasis, while the cingulate cortex is thought to be responsible for integrating emotional context with interoception (Ketai et al., 2016). DMN comprises three major subdivisions, including the ventromedial prefrontal cortex, posterior cingulate cortex, and precuneus (Greicius et al., 2003). Several lines of evidence have demonstrated that the DMN is associated with social cognition, introspection, prospection and memory (Dutta et al., 2019), and altered functional connectivity of the DMN is associated with cognitive decline (Sheline and Raichle, 2013). The SN is composed of the prefrontal cortex, insula, supramarginal cortex, and cingulate cortex, and SN is responsible for the detection of salient events, the switch of attention and control over behavior (Rosemann and Thiel, 2019). In the current study, the IN, DMN, and SN exhibited a decrease in activity following model establishment. Reduced functional activity was observed in the left visual cortex and right AcbSh. Previous studies demonstrated that MCAO resulted in retinal ischemia in rats, and Horner syndrome occurred ipsilateral to cerebral infarction and was represented in the ipsilateral visual cortex area (Kim et al., 2020). The AcbSh is an essential brain site for emotion- and motivation-related learning and memory (Huang et al., 2011). Alterations in brain functional networks may be related to functional dysfunction in rats with cerebral ischemia–reperfusion injury.

Electroacupuncture has been widely used as a component of traditional Chinese medicine for thousands of years. Different EA frequencies may result in different biological effects. High-frequency EA releases dynorphin to mediate the analgesic effects, while a low frequency releases endomorphin, encephalin, and endorphin, which may play an important role in recovery after stroke (Tian et al., 2016; Lee et al., 2020). Reportedly, EA exerts neuroprotective effects in the acute phase following ischemic stroke and may also facilitate functional recovery in the extended poststroke recovery phase (Liu et al., 2021). ST36 and LI11 are located near the knee and elbow joints, respectively, and are commonly applied in the treatment of ischemic stroke (Xie et al., 2013; Liu et al., 2016b). These two acupoints are considered sea points of the Yangming stomach meridian of the foot and the Yangming large intestine meridian of the hand, respectively. Based on traditional Chinese medicine theories, acupuncture at LI11 and ST36 could achieve a therapeutic effect by regulating the balance of qi and blood (Li et al., 2016). Behavioral evaluation is non-invasive and convenient and can be conducted continuously. The ladder rung walking test does not require painful aversive stimuli or other manipulation. The present results showed that EA treatment could decrease the foot fault rate of the affected limbs after MCAO/R. Acupuncture plays an important role in brain functional reorganization and compensation. The central nervous system may be differentially encoded after being triggered by acupuncture at different sites of the body, and the corresponding functional networks may mediate the specific therapeutic efficacy of acupuncture (Qin et al., 2011). Acupuncture at Yanglingquan (GB34) can regulate multiple brain networks in stroke patients with hemiplegia and likely transmit information between the cognitive network and SMN through the DMN as a relay station to integrate the effective connectivity network (Fu et al., 2017). Another study indicated that acupuncture at Waiguan (TE5) could increase the cooperation of the bilateral SMN in stroke patients (Chen et al., 2014). The results of the present study demonstrated functional disruption of the whole-brain network in MCAO/R rats and the modulatory effect of EA intervention. The SMN, IN, DMN, and SN were related to the therapeutic effect of EA on stroke recovery. The functional activity of the left visual cortex, right corpus callosum, AcbSh, somatosensory cortex, and motor cortex was significantly increased following EA treatment. The corpus callosum is the major brain commissure connecting the cerebral hemispheres and plays a key role in transferring motor, sensor and cognitive information between bilateral hemispheres (Revanna et al., 2018). The somatosensory and motor cortices are strongly modulated in relation to behavioral performance (Voudouris and Fiehler, 2021). Furthermore, the interactions between the IN and SN showed a significant association with motor function performance, suggesting that the IN and SN play an important mediating role during stroke rehabilitation.

Several limitations exist in the current study that should be noted. First, the sample size of this study was relatively small. A larger sample size is required to further confirm this conclusion in the future. Second, we only followed the animals in the acute stage. Further observation will be needed to determine its long-term outcomes, making our findings more convincing. Despite the aforementioned limitations, the current study, with the administration of EA, has identified changes in the brain network in the pathogenesis of ischemic stroke.

## Conclusion

In conclusion, the results of the present study add to the increasing evidence that EA is an effective therapeutic strategy for ischemic stroke. EA at the LI11 and ST36 acupoints was able to enhance the functional connectivity of the brain network and effectively promote the recovery of limb motor function. This might be one of the functional mechanisms by which EA exerts its protective effect in ischemic stroke.

## Data availability statement

The original contributions presented in the study are included in the article/supplementary material, further inquiries can be directed to the corresponding authors.

## Ethics statement

All animal experiments were approved by the Committee on Animal Care and Usage of Shanghai University of Traditional Chinese Medicine (Approval No. PZSHUTCM200110002).

## Author contributions

S-SL, HW, and J-JW collected the data and wrote the manuscript. X-XX and Y-WZ processed the data. X-YH, M-XZ, and C-LS revised the manuscript. J-GX designed the study. All authors reviewed the manuscript and approved the final version to be published.

## Footnotes

1
www.fil.ion.ucl.uk/spm

2
www.mricro.com

3
http://trendscenter.org/software/GIFT/

## References

[B1] Bajic D. Craig M. M. Mongerson C. R. L. Borsook D. Becerra L. (2017). Identifying rodent resting-state brain networks with independent component analysis. *Front. Neurosci.* 11:685. 10.3389/fnins.2017.00685 29311770 PMC5733053

[B2] Calhoun V. D. Adali T. Pearlson G. D. Pekar J. J. (2001). A method for making group inferences from functional MRI data using independent component analysis. *Hum. Brain Mapp.* 14 140–151. 10.1002/hbm.1048 11559959 PMC6871952

[B3] Chen J. Wang J. Huang Y. Lai X. Tang C. Yang J. (2014). Modulatory effect of acupuncture at Waiguan (TE5) on the functional connectivity of the central nervous system of patients with ischemic stroke in the left basal ganglia. *PLoS One* 9:e96777. 10.1371/journal.pone.0096777 24927275 PMC4057077

[B4] Chen L. Li C. Zhai J. Wang A. Song Q. Liu Y. (2016). Altered resting-state signals in patients with acute stroke in or under the thalamus. *Neurosci. Bull.* 32 585–590. 10.1007/s12264-016-0064-3 27664033 PMC5563835

[B5] Cheng H. J. Ng K. K. Qian X. Ji F. Lu Z. K. Teo W. P. (2021). Task-related brain functional network reconfigurations relate to motor recovery in chronic subcortical stroke. *Sci. Rep.* 11:8442. 10.1038/s41598-021-87789-5 33875691 PMC8055891

[B6] Dai X. J. Min Y. J. Gong H. H. Gao L. Wang S. Y. Zhou F. Q. (2012). [Evaluation of the post-effect of acupuncture at Sanyinjiao (SP 6) under sleep deprivation by resting-state amplitude of low-frequency fluctuation: a fMRI study]. *Zhongguo Zhen Jiu* 32 47–52. 22295826

[B7] Dutta A. Mckie S. Downey D. Thomas E. Juhasz G. Arnone D. (2019). Regional default mode network connectivity in major depressive disorder: modulation by acute intravenous citalopram. *Transl. Psychiatry* 9:116. 10.1038/s41398-019-0447-0 30877271 PMC6420575

[B8] Fu C. H. Li K. S. Ning Y. Z. Tan Z. J. Zhang Y. Liu H. W. (2017). Altered effective connectivity of resting state networks by acupuncture stimulation in stroke patients with left hemiplegia: a multivariate granger analysis. *Medicine* 96:e8897. 10.1097/MD.0000000000008897 29382021 PMC5709020

[B9] Greicius M. D. Krasnow B. Reiss A. L. Menon V. (2003). Functional connectivity in the resting brain: a network analysis of the default mode hypothesis. *Proc. Natl. Acad. Sci. U.S.A.* 100 253–258. 10.1073/pnas.0135058100 12506194 PMC140943

[B10] Guo L. Huang Z. Huang L. Liang J. Wang P. Zhao L. (2021). Surface-modified engineered exosomes attenuated cerebral ischemia/reperfusion injury by targeting the delivery of quercetin towards impaired neurons. *J. Nanobiotechnol.* 19:141. 10.1186/s12951-021-00879-4 34001136 PMC8130330

[B11] Han X. Jin H. Li K. Ning Y. Jiang L. Chen P. (2020). Acupuncture modulates disrupted whole-brain network after ischemic stroke: evidence based on graph theory analysis. *Neural Plast.* 2020:8838498. 10.1155/2020/8838498 32922447 PMC7453235

[B12] Hu M. Cheng H. J. Ji F. Chong J. S. X. Lu Z. Huang W. (2021). Brain functional changes in stroke following rehabilitation using brain-computer interface-assisted motor imagery with and without tDCS: a pilot study. *Front. Hum. Neurosci.* 15:692304. 10.3389/fnhum.2021.692304 34335210 PMC8322606

[B13] Huang X. Tong Y. Qi C. X. Dan H. D. Deng Q. Q. Shen Y. (2020). Large-scale neuronal network dysfunction in diabetic retinopathy. *Neural Plast.* 2020:6872508. 10.1155/2020/6872508 32399026 PMC7204201

[B14] Huang Y. H. Ishikawa M. Lee B. R. Nakanishi N. Schluter O. M. Dong Y. (2011). Searching for presynaptic NMDA receptors in the nucleus accumbens. *J. Neurosci.* 31 18453–18463. 10.1523/JNEUROSCI.3824-11.2011 22171047 PMC3277808

[B15] Hutchison R. M. Mirsattari S. M. Jones C. K. Gati J. S. Leung L. S. (2010). Functional networks in the anesthetized rat brain revealed by independent component analysis of resting-state FMRI. *J. Neurophysiol.* 103 3398–3406. 10.1152/jn.00141.2010 20410359

[B16] Jeong B. Choi J. Kim J. W. (2012). MRI study on the functional and spatial consistency of resting state-related independent components of the brain network. *Korean J. Radiol.* 13 265–274. 10.3348/kjr.2012.13.3.265 22563263 PMC3337862

[B17] Jiang G. Yin X. Li C. Li L. Zhao L. Evans A. C. (2015). The plasticity of brain gray matter and white matter following lower limb amputation. *Neural Plast.* 2015:823185. 10.1155/2015/823185 26587289 PMC4637496

[B18] Kajimura S. Masuda N. Lau J. K. L. Murayama K. (2020). Focused attention meditation changes the boundary and configuration of functional networks in the brain. *Sci. Rep.* 10:18426. 10.1038/s41598-020-75396-9 33116216 PMC7595086

[B19] Ketai L. H. Komesu Y. M. Dodd A. B. Rogers R. G. Ling J. M. Mayer A. R. (2016). Urgency urinary incontinence and the interoceptive network: a functional magnetic resonance imaging study. *Am. J. Obstet. Gynecol.* 215 449.e1–449.e17. 10.1016/j.ajog.2016.04.056 27173081 PMC5045785

[B20] Kim Y. H. Oh T. W. Park E. Yim N. H. Cho W. K. Ma J. Y. (2020). Neuroprotective effects of Acer palmatum thumb. leaf extract (KIOM-2015E) against ischemia/reperfusion-induced injury in the rat retina. *Mol. Vis.* 26 691–704. 33088173 PMC7553724

[B21] Lee D. Y. Jiu Y. R. Hsieh C. L. (2020). Electroacupuncture at Zusanli and at Neiguan characterized point specificity in the brain by metabolomic analysis. *Sci. Rep.* 10:10717. 10.1038/s41598-020-67766-0 32612281 PMC7329888

[B22] Li G. Han X. Gao W. Song Z. Zhao S. Sun F. (2020). Influence of EGR3 transfection on imaging and behavior in rats and therapeutic effect of risperidone in schizophrenia model. *Front. Psychiatry* 11:00787. 10.3389/fpsyt.2020.00787 33192626 PMC7542223

[B23] Li S. S. Hua X. Y. Zheng M. X. Wu J. J. Ma Z. Z. Xing X. X. (2022). Electroacupuncture treatment improves motor function and neurological outcomes after cerebral ischemia/reperfusion injury. *Neural Regen. Res.* 17 1545–1555. 10.4103/1673-5374.330617 34916440 PMC8771092

[B24] Li X. Liu Y. Zhang Q. Xiang N. He M. Zhong J. (2016). Effect of catgut implantation at acupoints for the treatment of allergic rhinitis: a randomized, sham-controlled trial. *BMC Complement. Altern. Med.* 16:454. 10.1186/s12906-016-1400-x 27829410 PMC5103331

[B25] Liang L. Hu R. Luo X. Feng B. Long W. Song R. (2020). Reduced complexity in stroke with motor deficits: a resting-state fMRI study. *Neuroscience* 434 35–43. 10.1016/j.neuroscience.2020.03.020 32194224

[B26] Liu L. Zhang Q. Li M. Wang N. Li C. Song D. (2021). Early post-stroke electroacupuncture promotes motor function recovery in post-ischemic rats by increasing the blood and brain irisin. *Neuropsychiatr. Dis. Treat.* 17 695–702. 10.2147/NDT.S290148 33688192 PMC7935344

[B27] Liu S. Y. Hsieh C. L. Wei T. S. Liu P. T. Chang Y. J. Li T. C. (2009). Acupuncture stimulation improves balance function in stroke patients: a single-blinded controlled, randomized study. *Am. J. Chin. Med.* 37 483–494. 10.1142/S0192415X09006990 19606509

[B28] Liu W. Shang G. Yang S. Huang J. Xue X. Lin Y. (2016a). Electroacupuncture protects against ischemic stroke by reducing autophagosome formation and inhibiting autophagy through the mTORC1-ULK1 complex-Beclin1 pathway. *Int. J. Mol. Med.* 37 309–318. 10.3892/ijmm.2015.2425 26647915 PMC4716798

[B29] Liu W. Wang X. Yang S. Huang J. Xue X. Zheng Y. (2016b). Electroacupunctre improves motor impairment via inhibition of microglia-mediated neuroinflammation in the sensorimotor cortex after ischemic stroke. *Life Sci.* 151 313–322. 10.1016/j.lfs.2016.01.045 26979777

[B30] Longa E. Z. Weinstein P. R. Carlson S. Cummins R. (1989). Reversible middle cerebral artery occlusion without craniectomy in rats. *Stroke* 20 84–91. 10.1161/01.STR.20.1.84 2643202

[B31] Lu Y. Jiang L. Li W. Qu M. Song Y. He X. (2017). Optogenetic inhibition of striatal neuronal activity improves the survival of transplanted neural stem cells and neurological outcomes after ischemic stroke in mice. *Stem Cells Int.* 2017:4364302. 10.1155/2017/4364302 29104593 PMC5618753

[B32] Lv Q. Xu G. Pan Y. Liu T. Liu X. Miao L. (2021). Effect of acupuncture on neuroplasticity of stroke patients with motor dysfunction: a meta-analysis of fMRI studies. *Neural Plast.* 2021:8841720. 10.1155/2021/8841720 34188677 PMC8192216

[B33] Metz G. A. Whishaw I. Q. (2002). Cortical and subcortical lesions impair skilled walking in the ladder rung walking test: a new task to evaluate fore- and hindlimb stepping, placing, and co-ordination. *J. Neurosci. Methods* 115 169–179. 10.1016/s0165-0270(02)00012-2 11992668

[B34] Qin W. Bai L. Dai J. Liu P. Dong M. Liu J. (2011). The temporal-spatial encoding of acupuncture effects in the brain. *Mol. Pain* 7:19. 10.1186/1744-8069-7-19 21429192 PMC3071327

[B35] Raichle M. E. (2011). The restless brain. *Brain Connect.* 1 3–12. 10.1089/brain.2011.0019 22432951 PMC3621343

[B36] Revanna K. G. Rajadurai V. S. Chandran S. (2018). Agenesis of the corpus callosum with interhemispheric cyst: clinical implications and outcome. *BMJ Case Rep.* 11:bcr2018227366. 10.1136/bcr-2018-227366 30567179 PMC6301757

[B37] Rosemann S. Thiel C. M. (2019). The effect of age-related hearing loss and listening effort on resting state connectivity. *Sci. Rep.* 9:2337. 10.1038/s41598-019-38816-z 30787339 PMC6382886

[B38] Saad Z. S. Reynolds R. C. Jo H. J. Gotts S. J. Chen G. Martin A. (2013). Correcting brain-wide correlation differences in resting-state FMRI. *Brain Connect.* 3 339–352. 10.1089/brain.2013.0156 23705677 PMC3749702

[B39] Sheline Y. I. Raichle M. E. (2013). Resting state functional connectivity in preclinical Alzheimer’s disease. *Biol. Psychiatry* 74 340–347. 10.1016/j.biopsych.2012.11.028 23290495 PMC3537262

[B40] Song X. W. Dong Z. Y. Long X. Y. Li S. F. Zuo X. N. Zhu C. Z. (2011). REST: a toolkit for resting-state functional magnetic resonance imaging data processing. *PLoS One* 6:e25031. 10.1371/journal.pone.0025031 21949842 PMC3176805

[B41] Tian G. H. Tao S. S. Chen M. T. Li Y. S. Li Y. P. Shang H. C. (2016). Electroacupuncture treatment alleviates central poststroke pain by inhibiting brain neuronal apoptosis and aberrant astrocyte activation. *Neural Plast.* 2016:1437148. 10.1155/2016/1437148 27774321 PMC5059615

[B42] Van Kesteren E. J. Kievit R. A. (2021). Exploratory factor analysis with structured residuals for brain network data. *Netw. Neurosci.* 5 1–27. 10.1162/netn_a_00162 33688604 PMC7935039

[B43] Voudouris D. Fiehler K. (2021). Dynamic temporal modulation of somatosensory processing during reaching. *Sci. Rep.* 11:1928. 10.1038/s41598-021-81156-0 33479355 PMC7820441

[B44] Wang C. J. Wu Y. Zhang Q. Yu K. W. Wang Y. Y. (2019). An enriched environment promotes synaptic plasticity and cognitive recovery after permanent middle cerebral artery occlusion in mice. *Neural Regen. Res.* 14 462–469. 10.4103/1673-5374.245470 30539814 PMC6334594

[B45] Wang W. Jiang B. Sun H. Ru X. Sun D. Wang L. (2017). Prevalence, incidence, and mortality of stroke in china: results from a nationwide population-based survey of 480 687 adults. *Circulation* 135 759–771. 10.1161/CIRCULATIONAHA.116.025250 28052979

[B46] Williamson P. C. Allman J. M. (2012). A framework for interpreting functional networks in schizophrenia. *Front. Hum. Neurosci.* 6:184. 10.3389/fnhum.2012.00184 22737116 PMC3380255

[B47] Xiang X. Wang S. Shao F. Fang J. Xu Y. Wang W. (2019). Electroacupuncture stimulation alleviates CFA-induced inflammatory pain via suppressing P2X3 expression. *Int. J. Mol. Sci.* 20:3248. 10.3390/ijms20133248 31269659 PMC6651287

[B48] Xie G. Yang S. Chen A. Lan L. Lin Z. Gao Y. (2013). Electroacupuncture at Quchi and Zusanli treats cerebral ischemia-reperfusion injury through activation of ERK signaling. *Exp. Ther. Med.* 5 1593–1597. 10.3892/etm.2013.1030 23837037 PMC3702718

[B49] Yeo B. T. Krienen F. M. Sepulcre J. Sabuncu M. R. Lashkari D. Hollinshead M. (2011). The organization of the human cerebral cortex estimated by intrinsic functional connectivity. *J. Neurophysiol.* 106 1125–1165. 10.1152/jn.00338.2011 21653723 PMC3174820

[B50] Zhang C. Liu X. Xu H. Hu G. Zhang X. Xie Z. (2020). Protopanaxadiol ginsenoside Rd protects against NMDA receptor-mediated excitotoxicity by attenuating calcineurin-regulated DAPK1 activity. *Sci. Rep.* 10:8078. 10.1038/s41598-020-64738-2 32415270 PMC7228936

[B51] Zhang J. Mu Y. Zhang Y. (2022). Effects of acupuncture and rehabilitation training on limb movement and living ability of patients with hemiplegia after stroke. *Behav. Neurol.* 2022:2032093. 10.1155/2022/2032093 35530165 PMC9072040

[B52] Zhao Y. Luo D. Ning Z. Rong J. Lao L. (2019). Electro-acupuncture ameliorated MPTP-induced parkinsonism in mice via TrkB neurotrophic signaling. *Front. Neurosci.* 13:496. 10.3389/fnins.2019.00496 31156376 PMC6528026

[B53] Zhao Z. Wu J. Fan M. Yin D. Tang C. Gong J. (2018). Altered intra- and inter-network functional coupling of resting-state networks associated with motor dysfunction in stroke. *Hum. Brain Mapp.* 39 3388–3397. 10.1002/hbm.24183 29691945 PMC6866540

